# Recruitment and reach in a school-based pediatric obesity intervention trial in rural areas

**DOI:** 10.3389/fpubh.2023.1181757

**Published:** 2023-06-01

**Authors:** Bethany Forseth, Brittany Lancaster, Megan Olalde, Christie A. Befort, Rebecca E. Swinburne Romine, Meredith L. Dreyer Gillette, Kelsey M. Dean, Eve-Lynn Nelson, Ann M. Davis

**Affiliations:** ^1^Center for Children's Healthy Lifestyles and Nutrition, Kansas City, MO, United States; ^2^Department of Pediatrics, University of Kansas Medical Center, Kansas City, KS, United States; ^3^Department of Population Health, University of Kansas Medical Center, Kansas City, KS, United States; ^4^Life Span Institute, University of Kansas, Lawrence, KS, United States; ^5^Department of Pediatrics, Children's Mercy Kansas City, Kansas City, MO, United States

**Keywords:** representativeness, dissemination and implementation, implementation science, RE-AIM evaluation framework, youth, children

## Abstract

**Introduction:**

The purpose of this study is to evaluate two recruitment strategies on schools and participant participation rates and representativeness (reach) within a pediatric obesity treatment trial tailored for families who live in rural areas.

**Methods:**

Recruitment of schools was evaluated based on their progress toward enrolling participants. Recruitment and reach of participants were evaluated using (1) participation rates and (2) representativeness of demographics and weight status of participants compared to eligible participants (who did not consent and enroll) and all students (regardless of eligibility). School recruitment, as well as participant recruitment and reach, were evaluated across recruitment methods comparing opt-in (i.e., caregivers agreed to allow their child to be screened for eligibility) vs. screen-first (i.e., all children screened for eligibility).

**Results:**

Of the 395 schools contacted, 34 schools (8.6%) expressed initial interest; of these, 27 (79%) proceeded to recruit participants, and 18 (53%) ultimately participated in the program. Of schools who initiated recruitment, 75% of schools using the opt-in method and 60% of schools using the screen-first method continued participation and were able to recruit a sufficient number of participants. The average participation rate (number of enrolled individuals divided by those who were eligible) from all 18 schools was 21.6%. This percentage was higher in schools using the screen-first method (average of 29.7%) compared to schools using the opt-in method (13.5%). Study participants were representative of the student population based on sex (female), race (White), and eligibility for free and reduced-price lunch. Study participants had higher body mass index (BMI) metrics (BMI, BMIz, and BMI%) than eligible non-participants.

**Conclusions:**

Schools using the opt-in recruitment were more likely to enroll at least 5 families and administer the intervention. However, the participation rate was higher in screen-first schools. The overall study sample was representative of the school demographics.

## 1. Introduction

Pediatric obesity contributes to lifelong chronic diseases (e.g., type 2 diabetes) and therefore is a critical concern that needs to be addressed (1). Children who live in rural areas demonstrate a 26% greater risk of obesity compared to their urban counterparts (2, 3). Adding further concern, rural areas lack resources and programs for pediatric obesity treatments (4–6). One option for providing obesity treatments to children living in rural areas is through their local schools which may increase treatment access for a large number of youth (7, 8).

While schools may be an optimal setting for pediatric obesity treatments, many aspects of program uptake by participants are unknown in rural school settings, including best recruitment methods, and representativeness of individuals who are ultimately consented and enrolled. Understanding these facets of program uptake and representativeness is important for future work regarding treatment of pediatric obesity in rural school settings and will aid in bringing evidence-based interventions to families who live in rural areas and are in need of these services. The Reach Effectiveness/Efficacy Adoption Implementation and Maintenance (RE-AIM) framework provides a guide to evaluate both internal and external validity and support the dissemination and implementation of evidence-based interventions into practice (9). Reach specifically supports external validity and generalizability of results by examining the uptake and representativeness of the participants compared to the eligible community or population (in this case, all the children in the school). While previous researchers have applied the RE-AIM framework to a school setting, gaps remain (10–12). First, none of these studies involve an empirically supported pediatric obesity treatment program in accordance with the United States Preventative Services Task Force (USPSTF) guidance (26 contact hours, parents and children participating, etc.) (13). Second, while recruitment strategies have been reported, few studies systematically evaluate which strategies are most effective and provide the most representative study sample in the school setting.

The purpose of the current study was to address these existing gaps in the literature by evaluating recruitment of schools and participants in an 18-site randomized clinical trial. Specifically, we examine success in recruitment of both schools and participants and participant representativeness across select demographics, comparing two common participant recruitment strategies: (1) caregivers were contacted to opt-in to have their child screened for the study (opt-in) and (2) all children within targeted grades underwent body mass index (BMI) screening and eligible participants were invited to the study (screen-first).

## 2. Methods

### 2.1. Study design, setting, and participant eligibility

Briefly, iAmHealthy is a two-arm cluster randomized pediatric obesity intervention (14) that follows expert committee guidelines (15), delivered to schools in rural Kansas. Eligible families were those with a child in 2nd−4th grade from a participating school who had a child body mass index (BMI) between the 85th and 99th percentile for age and sex. Exclusion criteria included children with significant limits to physical mobility, significant medical issue (e.g., cancer), cognitive impairments, developmental delays, or did not speak English. Only one child from each family could be enrolled in the study. Randomization occurred at the school-level after baseline data collection; the two interventions were (a) a family-based behavioral intervention where families met for group sessions via telehealth and (b) a control group that received educational newsletters. Within the intervention arm, caregiver/child dyads attended group and individualized health coaching sessions (~25 contact hours) over the course of 8 months. Caregiver/child dyads in the control group were sent newsletters throughout an 8-month period. The full intervention protocol is described in more detail in Davis et al. (14).

### 2.2. Recruitment

#### 2.2.1. Recruitment of schools

Schools were recruited between spring 2017—fall 2019. Eligible schools were in cities or counties with a population <20,000 individuals and served students in 2nd, 3rd, and/or 4th grade. Various recruitment methods were utilized to recruit schools including paper flyers to all nurses, gym teachers and principals at all eligible public elementary schools in the state of Kansas, listserv emails, and a partnership with a state level school nursing organization who assisted in contacting schools. Recruitment materials included brief information about the study and contact information for the research team. For a school to be a site, they needed to first respond with a letter of support for participation from a school administrator (e.g., principal or superintendent).

After enrollment, school personnel who supported the study (e.g., school nurses and physical education teachers) underwent a virtual training with the research team on the study protocol, research ethics, and informed consent. Then, school personnel were added to the study Institutional Review Board and were shipped equipment to screen, consent, and enroll students in 2nd−4th grade for the study. School personnel were required to recruit at least 5 families to be randomized as a site; if they were not able to recruit enough families, the school did not move forward to randomization. Throughout the onboarding process, researchers had regular communication with school personnel; therefore, reasons for non-inclusion in the study were gathered from school personnel during these onboarding meetings. School personnel were paid $30 per hour for hours they worked on the study, with a maximum of $2,000 per site.

#### 2.2.2. Participant recruitment strategies

School personnel recruited study participants within their schools. The two strategies to recruit participants were: (1) opt-in or (2) screen-first. School personnel self-selected which recruitment method they preferred based upon their school preference and history of conducting school-wide annual BMI screenings (i.e., they were not randomized to their recruitment method). Participant recruitment occurred after each school was recruited and occurred between fall 2017—spring 2020.

##### 2.2.2.1. Opt-in

For this recruitment method, school personnel contacted caregivers via personal calls, emails, and flyers, and caregivers opted in for their child to have a BMI screening to participate in a new research program available at their school that was designed to “help rural children in our state to live healthier lives”. Researchers did not formally track contact methods or number of contact attempts, but school personnel were encouraged to reach out to caregivers using multiple communication methods. Brief information on what study participation would involve was included as well as the treatment options (i.e., randomization to one of two intervention groups) and a link to the study website. Caregivers who expressed interest gave permission for their child to be assessed for eligibility. Specifically, height and weight data were collected by the school personnel with BMI calculation assistance provided by the study team (if needed). Once BMI eligibility was established, families were screened for other inclusion/exclusion criteria and if eligible, invited to consent and enroll.

##### 2.2.2.2. Screen-first

For this recruitment method, school personnel measured the height and weight of all students in 2nd−4th grade, which they were already doing as part of an annual BMI screening program. The study team offered assistance for BMI calculation (if needed). The number of students screened and the number of students eligible were provided to the research team. Caregivers of children who were eligible based on BMI criteria were then provided information about the study by school personnel via personal calls, emails, and flyers. Once contacted, eligible children were then screened for other inclusion/exclusion criteria and subsequently invited to participate.

### 2.3. Measures

#### 2.3.1. Participation rates

Regarding participation rate, we report total number of eligible children in the school (based on grade and BMI), total number enrolled, and participation rate (number enrolled divided by the number eligible). For schools using screen-first, the number of children eligible is based on measurements taken on all students in the 2nd−4th grades. For schools using opt-in, we estimated the number of eligible students by taking the total number of children in 2nd−4th grade (from Kansas State Department of Education) and multiplying it by 35.4% (the average percentage of children with overweight/obesity in screen-first schools from this study). We used 35.4% rather than the pediatric rate for overweight and for Kansas (29.5%) (United Health Foundation) (16) because it is more specific to these rural communities participating in the current study. For descriptive purposes, we also report the number eligible from those who opted to be screened at schools using the opt-in method.

#### 2.3.2. Representativeness of sample

For this outcome, we compared participants to non-participants on the following: BMI metrics, sex (female), race (White), and eligibility for free/reduced-priced lunch. Demographic characteristics of the school were collected from the Kansas State Department of Education website, which includes school and classroom-level information (17). In accordance with the Family Educational Rights and Privacy Act (FERPA), the Kansas State Department of Education does not provide data if <10 children fall within a given category; for this reason, grade or classroom level counts of free and reduced-price lunch were limited. When limited, the percentage of students eligible for free/reduced-price lunch from the whole school was applied to the students in 2nd−4th grade. No other race category besides White was selected because there was limited information due to FERPA reporting standards in these communities. Four schools had limited sex or race data and were excluded from these analyses as school-wide data was not available.

With respect to BMI metric comparisons, height and weight data provided by school personnel were utilized to make comparisons on body mass index (BMI), BMI z-score (BMIz), and BMI percentile (BMI%) between children who were enrolled in the study and those who were eligible but did not enroll.

### 2.4. Statistical analysis

Recruitment of schools is reported at each stage of participation. Frequencies and percentages are reported for child participation rates by school and by recruitment strategy. A Pearson correlation was used to examine the relationship between child participation rate and school size. Paired *t*-test analyses were used to evaluate the representativeness of demographics between all students in the 2nd−4th grade classrooms and study participants at the school level. Additionally, paired *t*-test analyses were used to compare the representativeness of BMI data between eligible individuals (based on BMI screening) and study participants.

## 3. Results

### 3.1. Recruitment of schools

**Figure 1:**
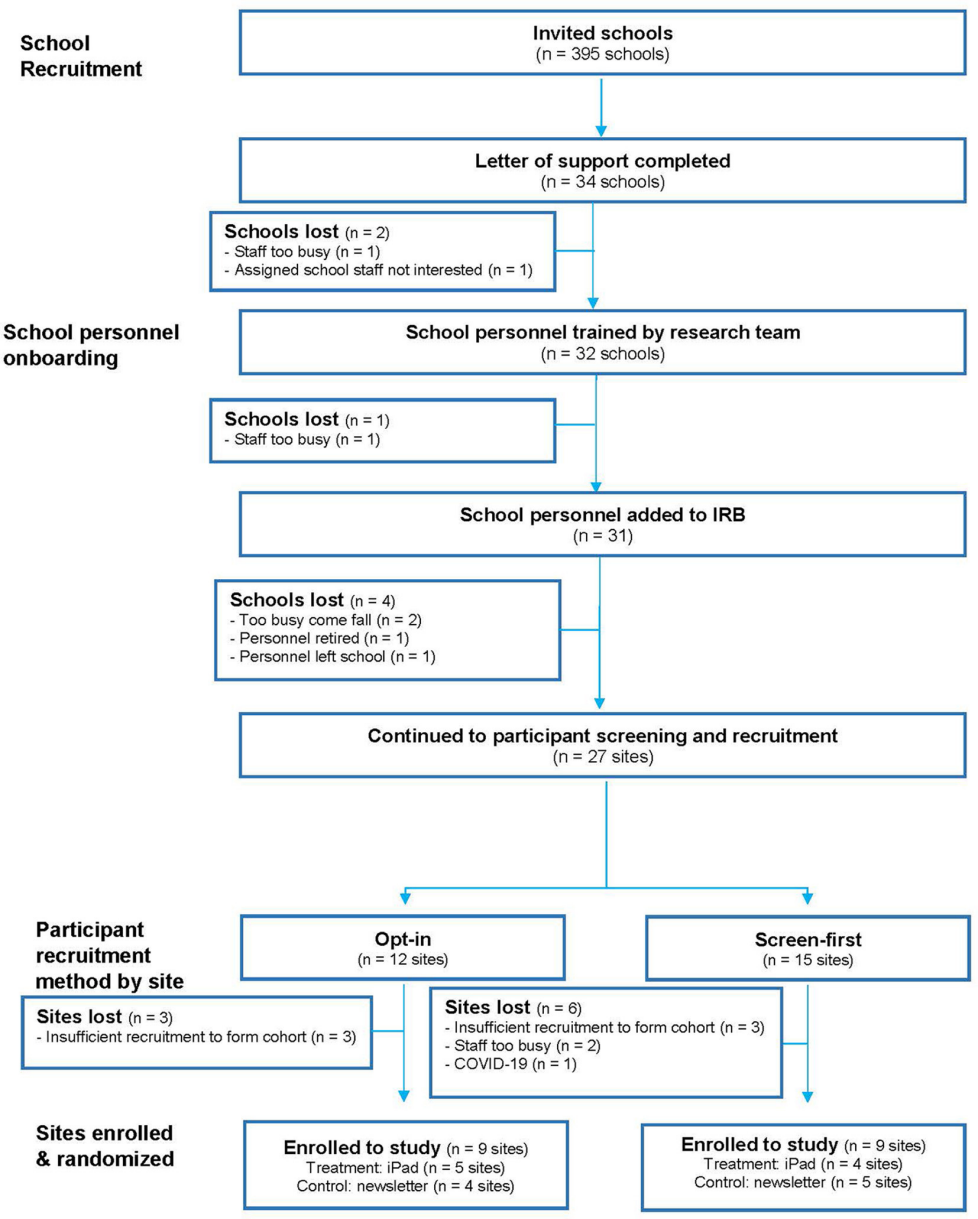
Recruitment of schools.

The research team invited 395 elementary schools in rural Kansas to participate (Figure 1). Of the schools contacted, 34 expressed interest. Twenty-seven schools began participant recruitment and 18 schools (52.9% of those interested; 4.5% of total contacted schools) enrolled sufficient participants to continue to randomization (Figure 1). Enrolled schools were recruited via flyers (*n* = 12), listserv emails (*n* = 1), direct contact with a state nursing organization (*n* = 2), or referral from a current participant or local nurse (*n* = 3). To increase recruitment, the study team directly called 42 eligible schools, but no schools were enrolled through this method. Schools that were interested but did not participate in the program commonly listed school personnel being too busy and insufficient recruitment as reasons why they were unable to move forward with the study.

### 3.2. Participant recruitment strategies

From the 27 schools who initiated recruitment of participants, nine were not able to successfully recruit at least 5 families and discontinued their involvement: this included 3 of 12 (25%) schools using the opt-in method and 6 of 15 (40%) schools using the screen-first method (Figure 1). Two of the twelve schools within the opt-in recruitment strategy that successfully recruited participants reported using additional tactics to increase their response rates. At one school, the school personnel hosted a table at a back-to-school night that allowed them to talk to most caregivers. The second school offered prizes for returning an opt-in form regardless of caregiver response.

### 3.3. Participation rates

**Figure 2:**
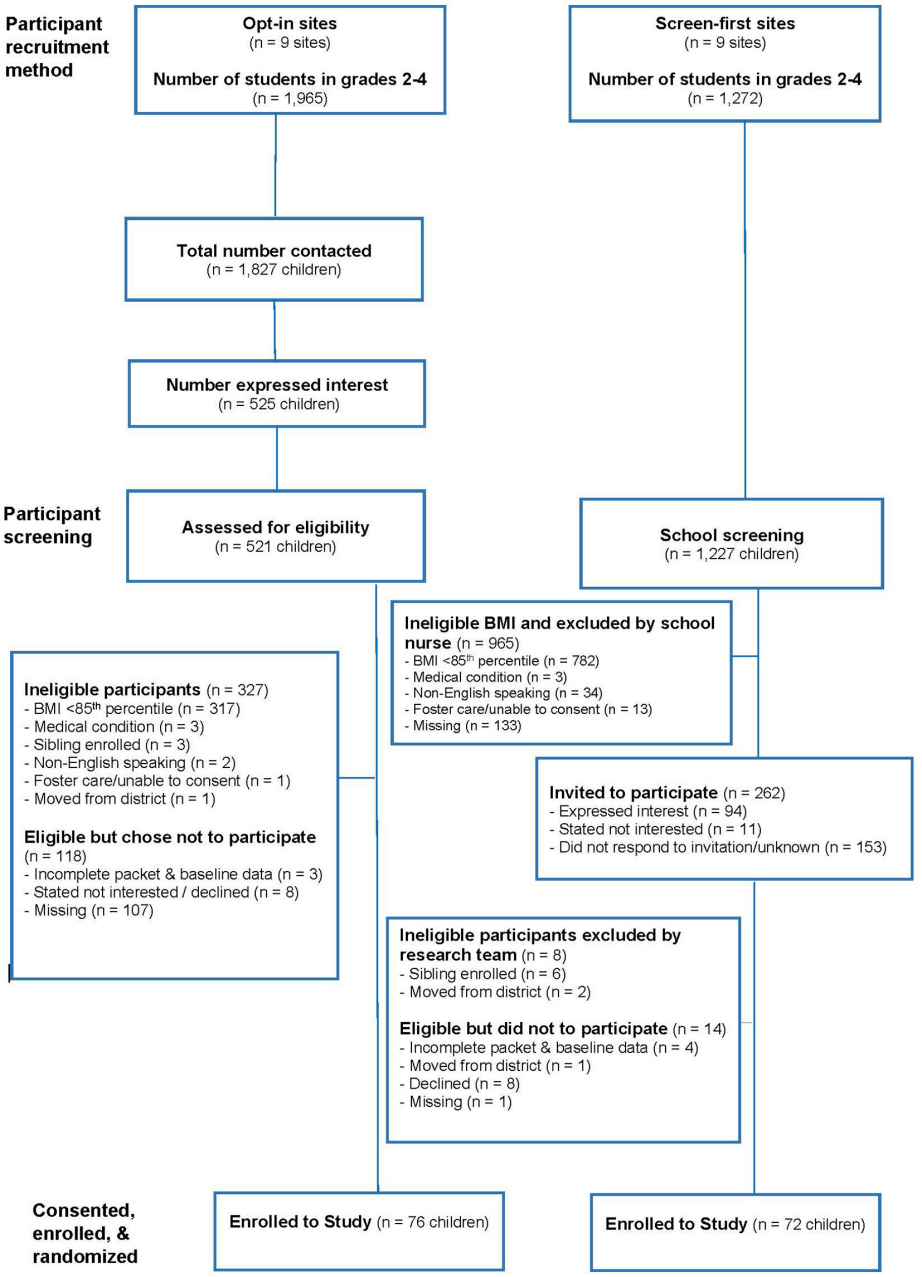
Recruitment of participants.

Figure 2 displays participant recruitment, screening, eligibility and enrollment by recruitment method. The participation rate from all 18 schools in the study was 21.6%. Excluding the schools in each recruitment method that were unable to recruit at least 5 families, the participation rate was higher in schools using the screen-first method (average of 29.7% across 9 schools) compared to schools using the opt-in method (average of 13.5% across 9 schools). Table 1 displays participation rates based on recruitment method by school; participation rates ranged from 5.6 to 46.7% across all schools. There was a significant, negative relationship between school size and participation rate (*r* = −0.48, *p* = 0.044), with smaller schools having higher participation rates on average.

**Table 1:** Eligibility and participation rate by recruitment method and by school.

**Recruitment method**	**School ID**	**Students in grades 2–4**	**Number screened**	**Number eligible from screening**	**Estimate of number eligible**	**Total number consented and enrolled**	Recruitment rate (%)^*^
Opt-in flier	A	104	50	-	37	7	19.0
	B	80	78	-	28	8	28.2
	D	356	61	-	126	7	5.6
	E	178	46	-	63	7	11.1
	G	172	62	-	61	9	14.8
	H	357	60	-	126	11	8.7
	I	152	18	-	53	6	11.5
	L	145	8	-	51	7	13.6
	N	421	141	-	149	14	9.4
	**Opt-in total**	**1,965**	**524**	**-**	**694**	**76**	
Screen-first	F	123	118	50	-	6	12.0
	J	71	73	20	-	7	35.0
	K	120	103	30	-	14	46.7
	M^†^	176	81	47	-	11	23.4
	O^‡^	162	160	12	-	9	75.0
	P	192	193	70	-	6	8.6
	Q	33	33	11	-	5	45.5
	R	207	204	94	-	7	7.4
	S	188	123	51	-	7	13.7
	**Screen-first total**	**1,272**	**1,088**	**385**	**-**	**72**	

* Recruitment rate = total # enrolled/estimate # eligible (opt-in) or = total # enrolled / # eligible from screening.

† Only screened 3rd and 4th graders.

‡ Screened kindergarten−4th grade.

### 3.4. Representativeness of sample

Table 2 shows representativeness of the study participants compared to the school student population for sex, race, and free and reduced-price lunch status. Overall, study participants were comparable to the student population regarding these characteristics. When examining sample representativeness within each recruitment method, the screen-first method resulted in a higher percentage of females in the study compared to the school population [68.4 vs. 50.9%; *t*_(6)_ = 2.895, *p* = 0.028].

**Table 2:** Comparison between study participants and students in grades 2–4 at participating schools (*n* = 18 sites).

	**Overall study**			**Opt-in schools**			**Screen-first schools**		
**Demographic characteristic**	**Student population**	**Participants**	***p*** **-value**	**Student population**	**Participants**	***p*** **-value**	**Student population**	**Participants**	***p*** **-value**
Children	3,237	148		1,965	76		1,272	72	
Females^a^	1,409 (49.3%)	79 (58.7%)	0.059	926 (48.0%)	40 (51.2%)	0.619	**591 (50.9%)**	**39 (68.4%)**	**0.028**
White^b^	2,079 (76.2%)	87 (71.8%)	0.300	1,339 (78.1%)	51 (77.1%)	0.837	680 (73.7%)	37 (64.7%)	0.271
Eligible for FRPL	1,821 (49.5%)	72 (53.9%)	0.411	1,128 (52.8%)	35 (44.5%)	0.251	693 (55.0%)	37 (54.5%)	0.957

Frequency (percentage). FRPL, free-or reduced price lunch.

a Overall study n = 16 sites, total participants n = 136 opt-in: participants n = 76 n = 9 sites, screen first: participants n = 60, sites n= 7.

b Overall study n = 16 sites, total participants n = 121, opt-in: participants n = 68, sites n = 8, screen first: participants n = 53, sites n = 6; bolded values signify p < 0.05.

Sixty percent of the schools sent researchers de-identified height and weight data for the study team to calculate BMI; thus, BMI data were available on all screened individuals for 13 of the 18 participating schools (missing data from 2 schools using opt-in recruitment and 3 schools using screen-first recruitment). Table 3 shows the representativeness of study participants compared to eligible individuals on BMI, BMIz, and BMI percentile (BMI%). Results indicate a significant, but small, difference between eligible individuals and study participants on BMI (23.0 vs. 24.2 kg/m^2^), BMIz (1.8 vs. 1.9), and BMI% (94.9 vs. 95.7th%), with the participant group having higher BMI metrics than the eligible individuals. When examining BMI representativeness based on recruitment method, both methods indicate significant differences between eligible individuals and study participants on BMI. Additionally, screen-first schools observed differences for BMIz, with participants having higher BMI metrics than eligible individuals.

**Table 3:** Representativeness of study sample compared to screened non-participants based on body weight measures.

	**Overall study**				**Opt-in schools**				**Screen-first schools**			
	**Screened**	**Eligible individuals**	**Participants**	***p*** **-value**	**Screened**	**Eligible individuals**	**Participants**	***p*** **-value**	**Screened**	**Eligible individuals**	**Participants**	***p*** **-value**
Number of children	1,152	444	111		493	182	63		659	262	48	
Average BMI (kg/m^2^)	18.8 (4.3)	**23.0 (4.2)**	**24.2 (4.3)**	**0.002**	18.6 (4.1)	**22.7 (4.0)**	**23.7 (4.3)**	**0.044**	19.0 (4.5)	**23.3 (4.3)**	**24.9 (4.5)**	**0.025**
Average BMIz	0.7 (1.1)	**1.8 (0.5)**	**1.9 (0.5)**	**0.01**	0.6 (1.1)	1.8 (0.5)	1.9 (0.5)	0.200	0.7 (1.1)	**1.8 (0.5)**	**2.0 (0.5)**	**0.027**
Average BMI%	66.9 (28.6)	**94.9 (4.3%)**	**95.7 (4.3)**	**0.044**	66.8 (28.2)	94.7 (4.3)	95.3 (4.7)	0.384	67.1 (28.9)	95.0 (4.2)	96.3 (3.7)	0.079

Mean (standard deviation) or frequency (percentage; %); p-values for t-test comparisons between eligible individuals and study participants^;^ bolded values signify p < 0.05.

## 4. Discussion

This study examined recruitment of schools, participant recruitment strategies, and representativeness of study participants in a pediatric obesity treatment tailored for families living in rural areas.

The most effective method to recruit schools was by mailing out flyers; however, this study enrolled only 4.5% of the schools contacted. Compared to prior studies evaluating the success rate of school recruitment (18–20), both initial interest and school enrollment for this study was considerably lower, even when compared to a study examining rural school recruitment (21). Notably, each of these prior studies were obesity prevention programs. This may suggest that schools are more likely to participate in school-wide health promotion study rather than treatment programs for children within a certain BMI classification, which was the case in the present study. Further, the method to recruit schools in each of these studies was variable, with the prior studies utilizing partnerships with the U.S. Department of Education (21) and direct phone calls to teachers with pre-established relationships (19). Future studies should continue to evaluate the specific designs that lead to school enrollment to better identify effective methods.

With respect to recruitment of participants, significant variability in the participation rates existed based on schools and recruitment strategy. Schools who utilized the screen-first method were more likely to discontinue participation prior to beginning the intervention, possibly because this method does not consider interest in study participation. However, the screen-first method yielded a higher participation rate. With increasing numbers of school districts including health screening assessments (including BMI) (22) as part of yearly evaluations, this initial screening may be less of a burden on staff for future studies. In contrast, opt-in already takes participation interest into account but not all who are interested may be eligible for the study. Each recruitment method provides information on eligibility or interest, but neither method provides information on both. Therefore, future studies that deem interest is more important should use opt-in methods while studies that deem eligibility is more important should proceed with screen-first.

Overall, participation rates in this study were lower compared to prior pediatric obesity trials (22–72% recruitment rate) (23–26). Previous studies note recruitment challenges (23, 24) and occasionally need to modify recruitment strategies to attain the necessary sample size (24), however few assess best methods for recruitment, especially within school-based studies. Studies within clinics, observe that active recruitment strategies (e.g., physician referral or directed mailings) result in a higher number of enrolled participants compared to passive strategies (posted flyers and media advertisements) (26, 27). Interestingly, in a pediatric obesity treatment through clinics, researchers noted that participants enrolled through passive recruitment strategies had better retention than those enrolled through active recruitment strategies (26). These findings on recruitment strategies within clinics are similar to the present study with more active or targeted recruitment (i.e., screen-first) having a higher recruitment rate compared to the more passive (i.e., opt-in) recruitment strategy (29.7 vs. 13.5%, respectively). Anecdotally, while researchers did not formally track contact methods made by school personnel, it appears that school personnel who made direct phone calls to families seemed to be the most successful at recruiting, consenting, and enrolling participants. Additionally, smaller schools were more successful with recruitment compared to larger schools. This higher recruitment rate may be due to a variety of reasons such as it being easier for school personnel to contact a smaller pool of participants, school personnel having closer relationships with families, or families not being flooded with flyers on other school information (e.g., fewer resources).

Study participants were comparable to the student population in regard to percent female, percent White, and percent eligible for free and reduced-price lunch, and there were limited differences between recruitment methods. The exception is the higher percentage of recruited females from the screen-first recruitment method. This suggests that both recruitment methods are acceptable methods to help ensure that youth from all backgrounds are recruited to participate. With respect to weight status, participants who enrolled in our intervention had a higher weight status compared to all eligible. This may be due to a larger perceived need of the intervention. This provides support and emphasizes a need for prevention and health behavior improvement opportunities at the school level.

### 4.1. Recommendations and future considerations

Several recommendations can be offered for future school-based intervention research studies based on the current research. First, we recommend discussing pros and cons of each recruitment strategy with school personnel to help them determine the best option for their situation. If a school is not currently screening all youth for other reasons, this may put too much burden on school personnel. Anecdotally, there were many interested families who had children with a BMI within the healthy weight range who were captured within the opt-in method. This interest provides support for school-wide programs focused on obesity prevention rather than obesity treatments. Regardless of method, additional anecdotal information observes that personal contact with eligible families from a trusted contact (e.g., school nurse) appears to have been a very effective, albeit time intensive strategy for recruitment. With respect to recruitment of schools, recruitment efforts need to be flexible and active, especially after the initial wave of interest. Future multi-year studies may want to consider evaluating for differences in school personnel's engagement based on the method in which the school was recruited or how quickly they agreed to participate in the project. Anecdotally, the tenure of school personnel and number of schools they cover appeared to impact recruitment. This may be due to the impact of personal relationships and trust these professionals have with families. Thus, future research may want to more systematically examine the impact of tenure and school personnel workload. Finally, even though our study reports on children enrolled in the study and communication is sent through children, children are governed by their caregivers. Thus, there may be a disconnect in communication or children who are interested but their caregivers are not interested. This is an area to consider for future research.

A strength of this study is this is one of the first studies to examine different child recruitment strategies and representativeness of the study sample compared to the student population in rural schools. Another strength is the collection and reporting of data on BMI screenings for two thirds of the schools in the study. The use of FERPA data from the Kansas State Department of Education to characterize the representativeness of the study sample is both a strength and a weakness. A strength of this data is it provided local, school-specific, FERPA data at the grade level. Unfortunately, due to the privacy policy and small sizes of the grades, we were unable to compare races other than White (e.g., Black and multi-racial) or ethnicity (individuals identifying as Hispanic) between the student population and study participants. An additional limitation of the current project is that it was only provided in English, and many participants who were excluded for being non-English speaking identified as Spanish speakers. Thus, by only offering the program in English, we may have limited participation for some families who were Hispanic. Recruitment of schools took place prior to the COVID-19 pandemic and only one school was recruiting families during the start of the pandemic; findings from this study may not be generalizable to recruitment during a pandemic. Finally, school personnel self-selected their recruitment method, rather than being randomized to a method, and this self-selection may have impacted the schools' ability to recruit enough families and participate in the intervention.

## 5. Conclusion

This study examined recruitment of schools, participant recruitment strategies, and representativeness of study participants in a pediatric obesity treatment tailored for families living in rural areas. This study was the first to compare school's use of opt-in vs. screen-first recruitment methods for an obesity intervention trial.

Schools using the opt-in method were more likely to enroll at least 5 families and administer the program, but schools using the screen-first method had a higher participation rate. The utilization of a screen-first approach takes substantial effort on the part of school personnel, but ultimately allows for a more targeted approach to recruitment. For schools that already collect BMI data, utilizing the screen-first approach may result in less burden and greater participation rates. However, for those not already screening for BMI, it may be helpful to evaluate the amount of time school personnel is willing and able to dedicate to recruitment. Our findings suggested that both methods recruited a representative sample and thus are acceptable options moving forward.

## Data availability statement

The raw data supporting the conclusions of this article will be made available by the authors, without undue reservation.

## Ethics statement

The studies involving human participants were reviewed and approved by University of Kansas Medical Center Institutional Review Board. Written informed consent to participate in this study was provided by the participants' legal guardian/next of kin.

## Author contributions

BF led the overall study, conducted the data analysis and interpretation, and wrote and revised the manuscript. BL contributed to data analysis, data interpretation, and initial writing of the manuscript and manuscript edits. MO contributed to the data collection and interpretation and manuscript revisions. CB contributed to study design and manuscript edits. RS and MD contributed to data analysis and interpretation and manuscript edits. KD contributed to the data collection and interpretation and manuscript revisions. E-LN contributed to data collection and manuscript edits. AD contributed to study design, data collection, data analysis interpretation, and manuscript edits. All authors contributed to the article and approved the submitted version.
